# Heavy Metal Exposure: Molecular Pathways, Clinical Implications, and Protective Strategies

**DOI:** 10.3390/antiox13010076

**Published:** 2024-01-05

**Authors:** Hajime Koyama, Teru Kamogashira, Tatsuya Yamasoba

**Affiliations:** 1 Department of Otolaryngology and Head and Neck Surgery, Graduate School of Medicine, The University of Tokyo, Tokyo 113-8654, Japan; 2 Tokyo Teishin Hospital, Tokyo 102-0071, Japan

**Keywords:** heavy metal toxicity, aging, antioxidants, cellular damage

## Abstract

Heavy metals are often found in soil and can contaminate drinking water, posing a serious threat to human health. Molecular pathways and curation therapies for mitigating heavy metal toxicity have been studied for a long time. Recent studies on oxidative stress and aging have shown that the molecular foundation of cellular damage caused by heavy metals, namely, apoptosis, endoplasmic reticulum stress, and mitochondrial stress, share the same pathways as those involved in cellular senescence and aging. In recent aging studies, many types of heavy metal exposures have been used in both cellular and animal aging models. Chelation therapy is a traditional treatment for heavy metal toxicity. However, recently, various antioxidants have been found to be effective in treating heavy metal-induced damage, shifting the research focus to investigating the interplay between antioxidants and heavy metals. In this review, we introduce the molecular basis of heavy metal-induced cellular damage and its relationship with aging, summarize its clinical implications, and discuss antioxidants and other agents with protective effects against heavy metal damage.

## 1. Introduction

Heavy metals are those with a density >4.5 g/cm^3^ and include lead, chromium, cadmium, mercury, and arsenic [1]. Heavy metals are often found in soil and can contaminate drinking water, posing a serious threat to human health [2,3]. Heavy metals exist in natural sources such as volcanic eruption, natural deposits, or sea salt spray (lead), tectonic and hydrothermal events (chromium), dust storm or wildfire (cadmium), degassing or weathering of rock (mercury), and weathering of rock or microbial colonization (arsenic). People are exposed to heavy metals from mining, tanning, and textile dyeing (chromium), battery manufacturing (cadmium), pesticides and fertilizers (mercury), and smelting (arsenic) [4]. Heavy metal exposure damages many organs [5,6], and its effect in the prenatal stage is critical, especially during neural development [7]. Heavy metal toxicity depends on the absorbed dose, route of exposure, and duration of exposure—acute or chronic. Heavy metal toxicity can lead to various disorders and can result in excessive damage due to oxidative stress induced by free radical formation [8]. In this review, the molecular mechanisms underlying heavy metal-induced toxicity are discussed from the perspective of mitochondrial dysfunction related to oxidative stress and endoplasmic reticulum (ER) stress, including their interaction, as well as protective agents against heavy metal exposure.

## 2. Molecular Pathways in Heavy Metal-Induced Cytotoxicity and Relation with Aging

Heavy metals induce cytotoxicity by molecular mechanisms, including oxidative stress associated with mitochondrial dysfunction, apoptosis, necrosis, and ER stress, which are interconnected. Recent advances in systems biology and in vitro label-free proteomic approaches have revealed that metal exposure induces significant changes in protein expression during mitochondrial dysfunction, oxidative stress, ubiquitin proteome dysfunction, and mRNA splicing [9].

### 2.1. Oxidative Stress, Mitochondrial Stress, and Mitochondrial Dysfunction

Mitochondrial dysfunction is one of the main phenomena in heavy metal-induced cytotoxicity. Heavy metals can damage mitochondria both inside the organelle and on the organelle surface, and these mechanisms interact with each other.

#### 2.1.1. Heavy Metals Affect Cellular Redox Homeostasis (Figure 1 and Figure 2)

Normal cellular metabolism forms reactive oxygen species (ROS) and is controlled by antioxidant enzymes. An imbalance between ROS production and defense causes oxidative stress. Excessive ROS damages cells through three basic pathways: lipid peroxidation of membranes, oxidative modification of proteins, and DNA damage [10]. Lipids are abundant in cellular membranes, and lipid peroxidation by ROS causes membrane dysfunction, such as a decrease in membrane fluidity and an increase in membrane leakiness, leading to mutilation of membrane proteins, enzymes, and receptors. Proteins are also a target of ROS. ROS cause various post-translational protein modifications such as oxidation of sulfur-containing side chains, chlorination of side chain amines, oxidation of histidines and tryptophans, formation of dityrosine leading to oligomerization, fragmentation, destabilization, aggregation, and/or increased degradation of proteins [11]. DNA is susceptible to ROS attack, particularly guanine, which is easily oxidized to 8-hydroxyguanine and 8-hydroxy-2-deoxyguanosine. Such abnormalities lead to inappropriate protein formation and cell damage.

**Figure 1:**
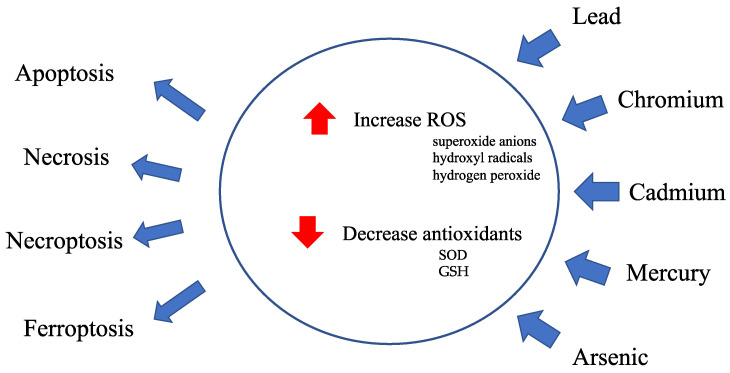
Overview of heavy metal toxicity. Heavy metal exposure causes a disruption of cellular redox homeostasis.

**Figure 2:**
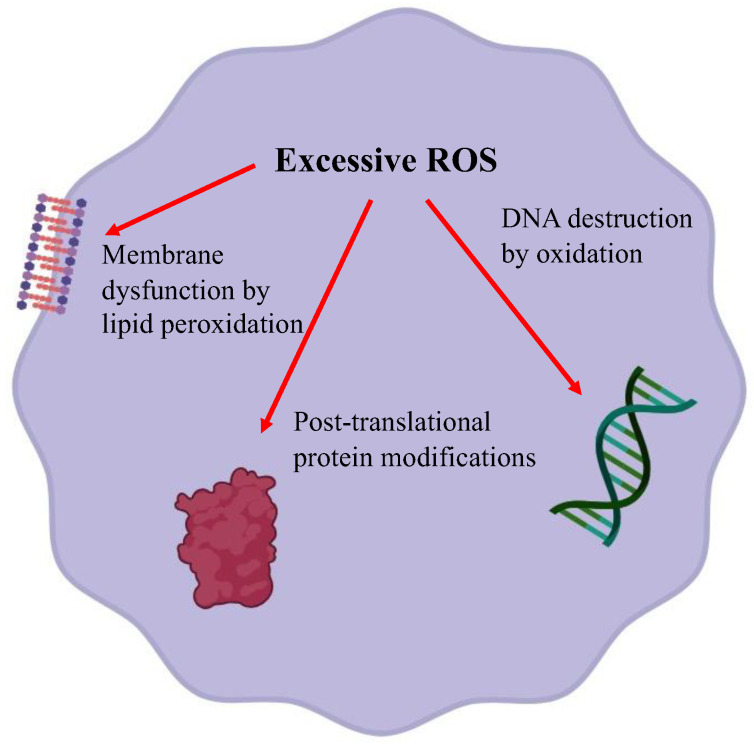
An effect of excessive reactive oxygen species on membrane lipid peroxidation, oxidative modification of proteins and DNA damage.

Antioxidants, including superoxide dismutase (SOD) and glutathione peroxidase, scavenge excessive ROS, such as superoxide anions, hydroxyl radicals, and hydrogen peroxide, produced in the mitochondria and protect against cellular damage from highly reactive ROS, preventing cellular damage caused by ROS. Heavy metals impair mitochondrial function by increasing ROS production and decreasing antioxidant activity.

ROS production is associated with heavy metal-induced mitochondrial damage. Increases in the expression of SOD1 and p62/Sequestosome 1 (SQSTM1) and an increase of cleaved caspase 3 are induced in the spleen following lead exposure, which corresponds to an increase in oxidative stress, apoptosis induction, and dysregulated autophagy [12]. In addition, lead increases ROS levels in the liver [13,14] and induces mitophagy via the phosphatase and tensin homolog (PTEN)-induced kinase 1 (PINK1)/Parkin pathway [15]. Lead damages the intracellular antioxidant system in hepatocytes [16] and blood [17], and an increase in ROS promotes DNA damage and inflammation [18,19,20] in hepatocytes [21,22], lungs [23], and plasma [24]. The increase in MDA levels upon Cd exposure suggests the promotion of lipid peroxidation by ROS in rat kidneys [25]. Mercury increases ROS production in erythrocytes [26] and neutrophils [27].

Heavy metals disturb the redox balance by decreasing antioxidant activity. Lead has the propensity to inhibit glutathione reductase, which converts oxidized glutathione (GSSG) to reduced glutathione (GSH) [28]. Cadmium damages mitochondrial function by reducing the activity of antioxidant enzymes. GSSG reductase activity is significantly decreased in the livers of cadmium-injected rats [29]. Cadmium also decreases SOD activity and GSH concentration in rat kidneys [25]. Mercury exposure dysregulates the oxidant detoxification system, thioredoxin1, and thioredoxin reductase1 redox system in neutrophils [27]. In summary, heavy metals disrupt the balance of antioxidants, increase harmful ROS, and impair mitochondrial function, disturbing cellular redox homeostasis.

Heavy metals affect the balance of the redox system through another pathway, ferroptosis. Ferroptosis was proposed as an iron-dependent form of non-apoptotic cell death in 2012 [30]. Ferroptosis is dependent on an intracellular level of free catalytically active iron [31]. Iron overload leads to the Fenton reaction, leading to overproduction of ROS as well as causing mitochondrial dysfunction by increased mitochondrial ROS production. ROS produced by iron overload results in cell death. The glutathione system plays a critical role in the regulation of ferroptosis. A key component is the Xc system, composed of SLC7A11 and SLC3A2, which facilitates the uptake of cystine while exporting cellular glutamate. Cystine is then converted to cysteine, an essential precursor for GSH synthesis, through the actions of GCL and GSS. The Xc system is a primary target of erastin, a ferroptosis inducer. Erastin not only interacts with the Xc system, but also binds to voltage-dependent anion channels 2 and 3 (VDAC2/3), leading to an increase in mitochondrial ROS generation [32]. GSH serves as a cofactor for the selenoprotein glutathione peroxidase 4 (GPX4), which plays a critical role in reducing lipid hydroperoxides to their corresponding alcohols [33]. These processes effectively prevent the harmful accumulation of lipoperoxidation products.

Arsenic increases cytoplasmic and mitochondrial iron accumulation through VDAC3 expression and down-regulation of SLC7A11 and GPX4 mRNA and protein expression in mouse brain and PC-12 cells [34]. It also induces ferroptosis in testicular cells, decreases GPX4, SLC7A11, Iron-Responsive Element-Binding Protein 2 (IREB2), and increases VDAC3 protein expression [35]. Cadmium induces ferroptosis in PC12 cells by increasing cellular iron content, repressing GPX4 expression [36]. This also happens in mouse seminiferous tubules and Leydig cells accompanying a significant decrease in GPX4 and an upregulation of SLC7A11 protein expression [37]. Mercury intoxication causes damage to rat astrocytes and rat liver cells through ferroptosis, with an increase in cytoplasmic ROS, lipid peroxidation, and decreased GPX4 activity [38]. Mercury also causes iron homeostasis. Hg exposure causes an increase free iron levels through heme degradation [39] or alteration of hypoxia-inducible factor-1α signaling [40], leading to ferroptosis. Lead exposure increases free iron levels in PC12 cells and causes ferroptosis [41]. Lead also affects SLC7A11 expression in neural stem cells [42] and inhibits GPX4 mRNA expression in chicken nerve tissue [43], all of which indicate ferroptosis.

#### 2.1.2. Heavy Metal Toxicity Affects the Mitochondrial Surface

The mitochondrial electron transport chain (mtETC) is located in the inner mitochondrial membrane and maintains mitochondrial respiratory functions. This is inhibited by the blockade of enzyme activity upon heavy metal exposure [44], which promotes the opening of mitochondrial permeability transition pores (mPTP). Mitochondrial dysfunction induces the apoptotic pathway, and mPTP regulates apoptosis and necrosis. This means that the maintenance of the mitochondrial membrane potential (mMP) and ROS by mPTP plays an important role in a molecular pathway of heavy metal-induced cytotoxicity. Mutations in mitochondrial DNA (mtDNA) induce various phenotypes related to aging and cancer, which can also be induced by heavy metal exposure [45,46]. Histologically, mitochondrial dysfunction is characterized by mitochondrial edema. Lead promotes mPTP opening and harms the mMP, leading to a decrease in ATP production in rat proximal tubular cells [47]. Chronic lead exposure leads to mitochondrial edema in mice hepatocytes [48]. Mercury induces the release of cytochrome c from mitochondria, followed by mPTP opening [49]. Cadmium and mercury induce neurotoxicity via mETC dysfunction, intracellular ROS formation, mMP decrease, and mPTP opening mediated by mPTP assembly in the rat neuronal cell line PC12 [50]. Mercury and cadmium inhibit the mitochondrial respiratory chain and rapidly dissipate mMP, promoting both necrosis and apoptosis, which cause cell death in rat hepatoma cells [51]. Cadmium leads to mPTP opening and causes mitochondrial dysfunction in the rat liver [52]. Chromium induces mitochondrial dysfunction and mitophagy mediated by ATF4 via ER stress [53]. In conclusion, heavy metal exposure adversely affects various components of the mitochondrial surface, disrupting the mtETC, promoting mPTP opening, and influencing the mMP, all of which contribute to mitochondrial dysfunction.

### 2.2. Heavy Metal Exposure Induces ER Stress and Mitophagy (Figure 3)

Heavy metal exposure triggers ER stress by accumulating unfolded proteins in the endoplasmic reticulum (ER) damaged by heavy metal binding; this is called the unfolded protein response (UPR). In the ER stress pathway, the misfolded proteins induced by toxic agents are detected by binding immunoglobulin protein (BiP)/glucose-regulating protein 78 (GRP78) and multiple signaling pathways [54]. Pathways, including the protein kinase R (PKR)-like endoplasmic reticulum kinase (PERK)-eukaryotic initiation factor 2α (eIF2α)-activating transcription factor 4 (ATF4) pathway, inositol-requiring enzyme 1α (IRE1α)-X-box binding protein 1 (XBP1) pathway [55] and activating transcription factor 6 (ATF6) pathway, are subsequently activated, leading to autophagy or mitophagy [56]. Mitophagy controls mitochondrial function by processing damaged mitochondria for digestion and maintenance of mitochondrial health. Lead specifically binds to GPR78, the major downstream target of the UPR induced by ER stress [57,58]. Cadmium also induces ER stress and ferroptosis [59]. Cadmium activates the PERK-eIF2α-ATF4 pathway and triggers autophagy activation [60,61]. Mercury induces GRP78 overexpression in rat glioma cells, and the effect of mercury on ER stress is dose-dependent [62]. Chromium induces GPR78 overexpression, inducing ER stress. Chromium activates the PERK-eIF2α-ATF6 pathway, fostering crosstalk between ER stress and mitochondrial dysfunction [53].

**Figure 3:**
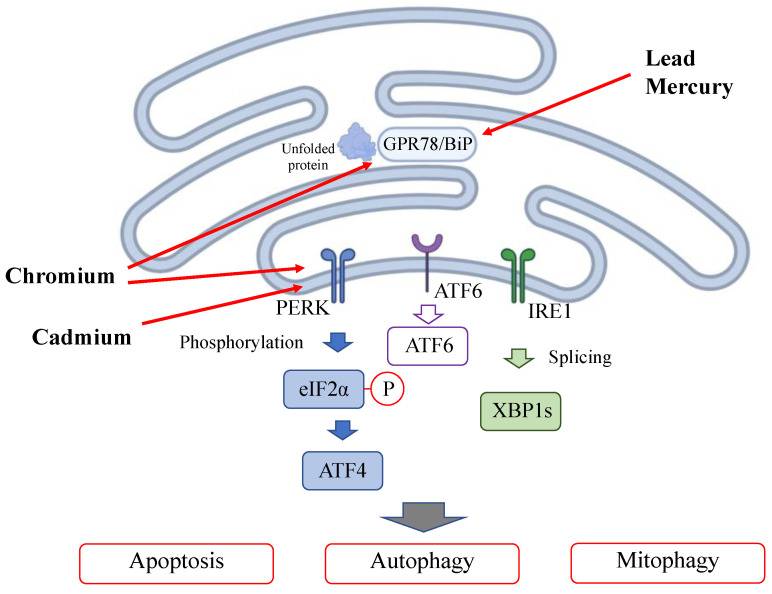
Effect of heavy metals on the endoplasmic reticulum.

### 2.3. Heavy Metal Exposure Induces Apoptosis, Necrosis, and Necroptosis

Heavy metal exposure induces cell toxicity and death via apoptosis, necrosis, and necroptosis, all of which are part of a molecular cascade of heavy metal cytotoxicity. During necroptosis, two members of the receptor-interacting protein kinase (RIPK) family, RIPK1 and RIPK3, are activated to phosphorylate mixed lineage kinase domain-like protein (MLKL), which compromises the cell membrane to execute cell death. Apoptosis and necrosis can occur simultaneously because the signaling pathways are interconnected [63,64]. ER stress activates apoptosis of cells [65,66], and ER stress pathways are also activated by necroptosis [67]. The difference between these pathways, apoptosis or necroptosis, depends on the cell type and is an important therapeutic target [68]. Lead acetate induces the overexpression of interleukin 6 (IL-6) in the brain and excessive activation of nerve cells, leading to necrosis [69,70]. High doses of lead acetate elevate the levels of caspase 8, caspase 9, and Bax in the brain, kidney, and liver [4], whereas a low dose of lead induces the apoptotic pathway in the brain [71]. Lead also activates necroptosis induced cell death in an olfactory cell line, elevating PIPK3 and MLKL [72]. Mercury induces the release of cytochrome c from mitochondria into the cytosol, which activates caspase 3 and induces apoptosis in the human leukemia cell line HL-60 [49]. Cadmium intake increases the expression of RIPK1, RIPK3, and MLKL to activate the RIPK3-dependent necroptotic pathway [73]. Cadmium exposure increases the levels of proapoptotic proteins (Bax, B-cell lymphoma 2 (Bcl-2), caspase 3, and caspase 9) and pronecropotic proteins (RIP, RIP3, and MLKL) [74,75]. Cadmium induces apoptosis at low concentrations in a dose-dependent manner and necrosis at high concentrations [76]. The effects of cadmium on apoptosis and necrosis differ during the cell cycle. Cadmium induces apoptosis in the G0 and S phases and necrosis in the S and M phases but hardly induces apoptosis in the G1 phase [77]. Chromium induces apoptosis via two major pathways: p53-dependent and independent pathways. Chromium leads to an increase in p53 protein levels in human lung epithelium and fibroblasts, leading to apoptosis [78,79]. Chromium induces apoptosis independently of p53 activation by causing mitochondrial instability, leading to capase-3 activation and subsequent damage to the mitochondria [80].

### 2.4. Heavy Metal Exposure Affects MicroRNAs (Figure 4)

Heavy metals induce alterations in microRNAs (miRNAs) that regulate the expression of various genes and signaling pathways [81]. miRNAs are non-coding, short, single-stranded RNAs (21–23 nucleotide-long); they regulate gene expression through RNA silencing and mitochondrial function and homeostasis, as well as modulate cell metabolism by targeting known oncogenes and tumor suppressor genes of metabolism-related signaling pathways [82]. Several human cancers are caused by the dysregulation of miRNA function. Recent studies have confirmed that miRNA dysregulation plays a role in carcinogenesis in many tissues [83]. The mRNA expression codons differ between the nucleus and mitochondria [84], and research on the cross-interaction of expression with miRNA found a new axis, including the regulation of mitochondrial function by miRNA [85,86]. Cadmium exposure causes miRNA overexpression and has negative effects on the kidneys and other organs [87]. Cadmium alters miRNA expression in the renal cortex. Cadmium induces the overexpression of 44 and suppresses the expression of 54 miRNAs [88]. Cadmium overload positively and negatively affected several miRNAs (185 miRNAs) in renal epithelial cells [89]. Of the miRNAs suppressed by cadmium induction, miR-125a and miR-125b function as anti-apoptotic elements by increasing the expression of Bcl2 and decreasing that of Bax, Bak, caspase-9, and caspase-3 [90]. Cadmium also alters miRNA expression in other organs. Cadmium decreases the expression of 12 miRNAs in liver cells (HepG2 cells) and impairs liver functions, such as lipid and fatty acid transportation, cholesterol metabolism, and fatty acid oxidation [91]. In the spleen, cadmium decreases miR-33-5p and increases AMPK, leading to AMPK-mediated autophagy [92]. Cadmium upregulated miR-101 and miR-144 in human bronchial epithelial cells and targeted cystic fibrosis transmembrane conductance regulator (CFTR), leading to respiratory damage [93]. miR-193, mi-R221, and miR-222 are upregulated in ovarian tissues exposed to cadmium [94], leading to apoptosis [95].

**Figure 4:**
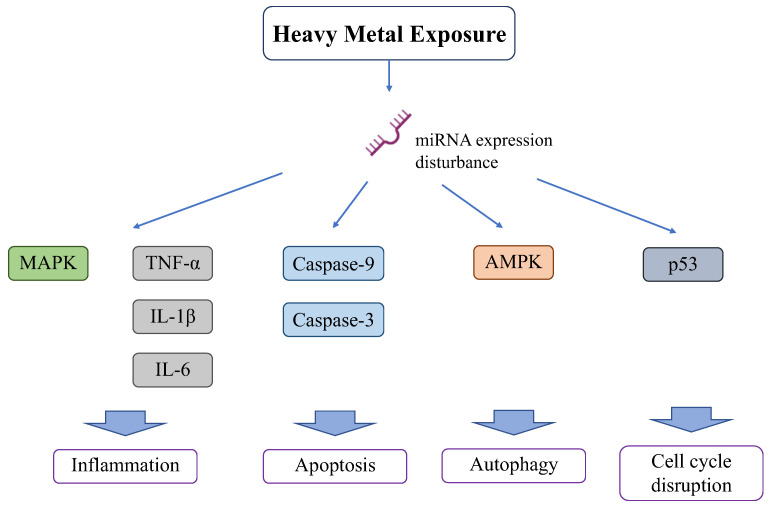
Effect of heavy metals on miRNA.

Other heavy metals also affect miRNAs and damage various organs. Lead promotes inflammation and apoptosis in mammary glands via the circRNA-05280/miR-146a/IRAK1 pathway [96]. Lead also increases miR-155 and activates inflammation in the MAPK pathway or production of inflammatory cytokines, such as TNF-α, IL-1β, and IL-6 [97].

### 2.5. Heavy Metal Exposure Affects Tumor and Senescence Pathways Involving p16/p21/p53

Heavy metal exposure impairs the p16/p21/p53 transcription pathway, which plays a critical role in cellular stress responses [98]. Heavy metal exposure alters the activation or inactivation of p53 both directly and indirectly through ROS [99]. Cadmium disrupts native p53 conformation, inhibits DNA binding, and downregulates the transcriptional activation of p21 in human breast cancer MCF7 cells [100]. Cadmium regulates p21/p53/p16 protein expression and mitochondrial dysfunction, leading to cellular senescence in bone marrow-derived mesenchymal stromal cells [101]. Long-term exposure to cadmium downregulates p16 via FNA hypermethylation in lymphocytes [102]. Lead exposure alters the promoter methylation rates of p16 in children [103]. Chromium exposure induces p16 methylation, decreases its expression, and damages lung cells [104]. Chromium increases CpG1 methylation levels of p16 and epigenetic modifications that damage human bronchial epithelial cells [105].

### 2.6. Relation with Aging

Aging is the process by which cells and organs cease to work properly or lose their function. Aging can be caused by similar mitochondrial dysfunction as occurs in heavy metal toxicity. ROS overproduction causes irregular signaling in mitochondria [106], acceleration of membrane instability by lipid peroxidation [107], and dysfunction of the DNA repair system [108], leading to malfunction of biological molecules and accumulation of irregular proteins and lipids. All this leads to cellular senescence [109]. 

Aging is also associated with ER stress. Accumulation of UPR plays a vital role in the cellular dysfunction which occurs in aging [110] These UPRs alter solubility, function inappropriately, and accumulate as plaques in various organs [111]

## 3. Pathophysiology of Heavy Metal-Induced Clinical Disorders

Considering the various pathways involved in heavy metal cytotoxicity, the pathophysiology of clinical disorders caused by heavy metal exposure involves several organs, exhibiting acute and chronic toxicity. In addition, complex interactions occur between each heavy metal, and their detrimental effects vary in different organisms. The new Bayesian network approach can be extended to incorporate information about metal–organism interactions [112]. A mixture of heavy metals results in an increased toxic effect compared with individual components at the same concentration [113].

### 3.1. Manifestation of Acute Heavy Metal Toxicity

The acute toxicity of heavy metal exposure induces various diseases and symptoms, including abdominal pain, anorexia, dyspepsia, diarrhea, fatigue, anxiety, numbness, memory and concentration difficulties, leukopenia, and thrombocytopenia.

Acute exposure to cadmium damages the respiratory system. Cadmium-containing nanoparticles accumulate in the lungs and penetrate the membranes of organelles, including mitochondria, damaging not only the lungs but also other organs [114]. One case report suggested that a patient with acute cadmium inhalation developed severe pneumonitis and died within 25 days [115].

Acute toxicity of arsenic causes gastrointestinal damage. Symptoms begin with a garlic-like taste, followed by dysphagia and severe vomiting. Acute paralytic syndrome occurs after the first symptom and ends with death within a few hours [116].

High-dose lead exposure causes acute hemolytic anemia. Lead inhibits the enzymes aminolevulinic acid synthetase (ALAS), aminolevulinic acid dehydratase (ALAD), and ferrochelatase, which are essential for heme synthesis, and their absence can cause anemia [117].

The acute toxicity of nickel originates from its combination with thiols, which results in the formation of Ni-thiol complexes [118]. These complexes generate free radicals that damage the body [119]. The clinical symptoms can be divided into two stages: immediate and delayed. Immediate effects include vomiting, irritation, headaches, and insomnia. Delayed effects include vertigo, palpitations, coughing, cyanosis, chest tightness, and lassitude [120]. 

Acute cadmium exposure can cause liver damage. Acute hepatotoxicity involves two pathways: the first, caused by the direct effects of cadmium, leads to initial injury, and the second causes subsequent injury due to inflammation. Primary injury appears to be caused by the binding of cadmium to sulfhydryl groups on critical mitochondrial molecules, causing oxidative stress, mitochondrial permeability transition, and mitochondrial dysfunction. Secondary injury is caused by the activation of Kupffer cells by triggering a cascade of events involving several types of liver cells and a large number of inflammatory and cytotoxic mediators [121].

### 3.2. Manifestation of Chronic Heavy Metal Toxicity (Figure 5)

Chronic toxicity due to heavy metal exposure affects a broad range of organs and manifests as neurological deterioration, cardiovascular diseases, reproductive problems, nephropathy, respiratory dysfunction, bronchitis, pulmonary edema, asthma, emphysema, hepatitis, anemia, hyperpigmentation, and cancer. In the following sections, we delve into each manifestation of chronic heavy metal toxicity.

**Figure 5:**
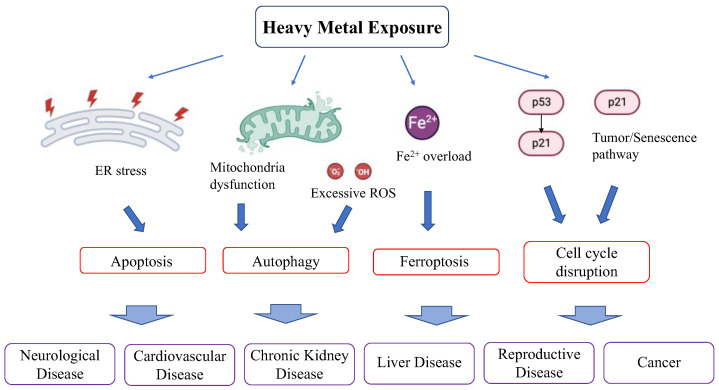
Effect of heavy metals on clinical disease.

#### 3.2.1. Neurological Symptoms

Heavy metals induce neurodegeneration through dyshomeostasis, ROS formation, and mitochondrial dysfunction [122]. Heavy metal exposure deteriorates mitochondrial quality. Calcium transporters in the mitochondrial membrane stabilize the mMP, and the disruption of calcium homeostasis triggers apoptosis in cells, which causes neurogenerative damage. Recent system biology analyses revealed that changes in protein expression associated with heavy metal exposure are related to neurodegenerative diseases, such as Parkinson’s disease and Alzheimer’s disease [9,123]. For example, PINK1/Parkin has a regulatory role in cleaning damaged mitochondria and underscores the importance of maintaining mitochondrial function in neural tissues, especially concerning Parkinson’s disease.

Among heavy metals, lead easily crosses the blood–brain barrier and acts as an alternative to calcium ions, which leads to interference with the normal action of calcium ions in the brain [124], affecting the uptake, release, and binding of GABA in the rat brain [125], calcium release through ryanodine receptors, and calcium signaling, thereby causing neurotoxicity in the rat brain [126].

Cadmium-induced oxidative stress has severe implications for neurological damage. ROS levels are increased in neuronal cell types, and mTOR and MAPK pathways are activated and are one of the causes of cytotoxicity in these cells [127]. Cadmium may be a risk factor for Alzheimer’s disease [128].

Mercury also damages neural cells. Methyl mercury is lipid-soluble and, therefore, easily crosses the blood-brain barrier. Mercury induces neurogenerative damage through oxidative stress and mitochondrial dysfunction, which can cause neurodegenerative diseases, such as Alzheimer’s disease [129,130] and amyotrophic lateral sclerosis [131]. Mercury is also considered a cause of Minamata disease, a neurodegenerative disease that occurs in Japan [132].

Heavy metals cause neuronal damage as individual metals, but mixtures of heavy metals also are harmful. Simultaneous exposure to lead, mercury, and cadmium causes greater brain damage in mice with reduced motor coordination and impaired learning and memory abilities than exposure to individual metals [133].

#### 3.2.2. Cardiovascular Symptoms

Heavy metal exposure induces hypertension through oxidative stress, impaired nitric oxide signaling, modified vascular response to neurotransmitters, disturbed vascular muscle Ca^2+^ signaling, renal damage, and interference with the renin-angiotensin system, whose mechanism involves multiple axes owing to the complexity of the vascular system [134]. Blood pressure regulation is related to calcium signaling and renal function. Mitochondrial dysfunction in the myocardial tissue triggers cardiovascular dysfunction.

Lead increases the levels of endothelin, norepinephrine, angiotensin-converting enzyme, and thromboxane [135], leading to increased blood pressure and organ hypoperfusion. An animal study suggested that lead intoxication results in hypertension, dyslipidemia, atherosclerosis, and cardiac complications [136]. In humans, chronic lead exposure is associated with hypertension [137]. A meta-analysis suggested that a two-fold increase in blood lead levels was associated with a rise of 1–1.25 mmHg in systolic blood pressure and 0.6 mmHg in diastolic blood pressure [138]. A human autopsy study revealed that lead exposure was associated with aortic atherosclerosis [139]. Elevated blood or bone lead levels are correlated with increased cardiovascular mortality [140].

Cadmium induces endothelial dysfunction and accelerates the formation of atherosclerotic plaques, which cause cardiovascular damage [141]. Cadmium exposure can also cause hypertension [142]. Cadmium exposure increases the risk of coronary heart disease, stroke, and peripheral arterial diseases [143].

#### 3.2.3. Chronic Kidney Disease

Heavy metal exposure leads to chronic kidney disease [144]. Lead accumulates in the kidney, and lead exposure inhibits glomerular development, resulting in renal dysfunction [145]. Chronic exposure to lead causes histopathological changes in the kidneys, such as progressive tubulointerstitial nephritis, characterized by the infiltration of leukocytes, interstitial fibrosis, and tubular atrophy [146]. Epidemiological studies have suggested that high serum levels of lead are associated with a higher risk of renal injury [147]. Furthermore, a high level of serum creatinine has been correlated with serum levels of lead [148].

Cadmium has a long half-life and tends to accumulate in the renal cortex for a long time [149]. Chronic exposure to cadmium has been associated with end-stage renal disease [150]. Cadmium also accumulates in the proximal tubules, causing Fanconi syndrome. Workers who work in cadmium-exposed areas tend to have kidney stones and tubulointerstitial nephritis [151].

Mercury induces neurological and nephrological damage. Mercury induces H_2_O_2_ formation and oxidative stress in rat kidney mitochondria [152] and causes glomerular and tubular dysfunction [153]. Arsenic also causes nephrological toxicity. Arsenic exposure induces ROS production in the kidneys and causes cellular damage and death [154]. An epidemiological study suggested that arsenic exposure is associated with albuminuria and proteinuria [155]. Arsenic in urine is associated with the prevalence of chronic kidney disease [156].

#### 3.2.4. Hepatitis

Heavy metals cause long-term damage to the liver. Cadmium accumulates in the liver and causes various metabolic disturbances [157], such as increases in hepatic, mitochondrial, and microsomal lipid peroxidation, as well as the depletion of glutathione [158].

Although its exact mechanism of action remains unknown, lead induces hepatotoxicity [48]. Lead accumulates in the liver and elevates aspartate aminotransferase (AST) and alanine aminotransferase (ALT) levels, leading to inflammation and hepatocyte death [20]. In India, lead-exposed individuals tend to have higher serum ASL, ALT, and bilirubin levels than healthy individuals [159], reflecting hepatotoxicity [160]. High-dose chromium exposure causes liver damage [161].

Arsenic destroys DNA, lipids, and proteins, damaging membranes, cells, and tissues in the liver [162]. Arsenic-intoxicated rat showed increased levels in thiobarbituric acid reactive substances, lipid hydroperoxides, protein carbonyl content and conjugated dienes, reduced DNA, decreased levels of the activities of membrane-bound ATPases, and increased levels of AST and ALT in the liver. Histological damage such as the extensive inflammation, dilated sinusoids, degeneration of hepatocytes with necrosis, vacuolization, and inflammatory cell infiltration also occurred. One study suggested that people who drink water containing more than 0.05 mg/L of arsenic show significantly more hepatomegaly than those who drink water containing less than 0.05 mg/L of arsenic [163]. 

#### 3.2.5. Reproductive Problems

Heavy metals can damage the reproductive system. They affect the male [164] and female reproductive systems [165] through various mechanisms.

Lead can bind to histidine in protamine and change the molecule’s conformation [166], resulting in sperm destabilization [167] and fertility issues. Lead can decrease testosterone levels [168], and the ROS produced by lead can cause severe damage to sperm quality and quantity [169,170]. Lead decreases the levels of several hormones, such as estradiol and LH, that are vital for reproduction [171]. Lead also elevates metallo-matrix proteins, which causes abnormalities in the placenta [172]; lead-exposed females have a high percentage of miscarriages [173].

Arsenic can damage the reproductive system. Arsenic decreases sperm number and mobility and affect sperm DNA [174,175]. Women exposed to arsenic experience disturbances in sex hormones and infertility [175].

Cadmium blocks calcium channels that play an important role in sperm fertilization [176]. Cadmium affects the transcription of a sperm protein (CatSper) [177] and damages sperm function [178]. It also decreases sperm mobility, causing infertility [179].

Mercury produces ROS, which causes infertility. Mercury decreases the number of sperms and alters their shape [179]. Mercury induces an imbalance in sex hormones [180], and low-dose mercury exposure causes stillbirth, spontaneous abortion, and infertility [181].

#### 3.2.6. Cancer

Heavy metals promote the production of ROS and chronic oxidative stress through various pathways including ferroptosis, which lay the groundwork conducive to carcinogenesis [182,183]. Three main mechanisms have been proposed to drive carcinogenesis: (1) disruption of cellular redox control, resulting in DNA damage, (2) suppression of essential DNA repair systems, resulting in genomic instability and accumulation of critical mutations; and (3) activation of oncogenic pathways and inactivation of tumor suppressors, disrupting the balance between cell proliferation and death [184].

Arsenic is a carcinogenic agent that induces oxidative DNA damage. It causes ROS production and breaks single-stranded and double-stranded DNA, forming 8-hydroxy-2′-deoxyguanosine (8-OHdG), which causes nucleotide conversion and tumor formation [185]. Bowen’s carcinoma is caused by arsenic exposure [186]; some lung tumors have also been reported to be associated with arsenic toxicity [187].

Cadmium has been implicated as a carcinogen and manifests its effects through several pathways. Cadmium produces ROS and increases the expression of c-Fos and c-Jun, which promote cancer [188]. Cadmium inhibits vital DNA mismatch repair systems and increases genomic instability, resulting in oncogenesis [189]. Cadmium can cause prostate cancer [190] and its inhalation can cause renal cancer [191].

Transcriptome analysis revealed that arsenic and cadmium exposure altered 167 genes which correlate with tumorigenesis, cell cycle, apoptosis, and oxidative stress in human lymphoblastoid cells [192]. Another study revealed that low-dose cadmium altered several gene expressions associated with maintaining cellular redox homeostasis such as increasing glutathione synthesis and antioxidant capacity, facilitating the survival or death response, and repairing damage or stimulating degradation [193].

## 4. Drugs for Protection against Heavy Metal-Induced Injury and Their Molecular Mechanisms

Chelating therapy is traditionally used to treat heavy metal toxicity. Moreover, while antioxidants are not generally used clinically, recent advances in molecular pathways have revealed that new compounds targeting oxidative stress effectively protect cells from heavy metal-induced cell damage (Table 1).

There are two types of protective drugs in terms of timing of use. Post-treatment drugs are used after exposure to heavy metals, whereas pretreatment intake is considered before exposure. Chelating agents and stoichiometric antioxidants are used for post-treatment strategies, and some antioxidants are used for pretreatment.

### 4.1. Posttreatment Drugs

#### 4.1.1. Chelating Agents

Chelation therapy is the primary treatment used to reduce the toxic effects of heavy metals. Chelating agents bind toxic metal ions and form complex structures that are easily excreted from the body, removing them from intracellular and extracellular spaces [194]. Chelators mobilize metals in tissues that mediate their excretion through the kidneys in the urine or the liver in bile. Concerns regarding enterohepatic recirculation and renal reabsorption of heavy metals are overcome by the use of lipophilic chelators, which can excrete larger amounts of heavy metals than aqueous chelators via bile; this is because aqueous chelators facilitate transport within the blood and excretion via the kidney, whereas lipophilic chelators exhibit greater penetration of cellular membranes, including those within the central nervous system, to chelate intracellular elements [195]. The major chelating agents reported to be effective against heavy metal toxicity are British anti-Lewisite (BAL), dimercaptopropane-l-sulphonate (DMPS), meso-2,3-dimercaptosuccinic acid (DMSA), sodium 2,3, monoisoamyl DMSA (MiADMSA), monomethyl DMSA (MmDMSA), monocyclohexyl DMSA (MchDMSA), calcium disodium ethylenediamine tetraacetic acid (CaNa2EDTA), calcium trisodium diethylenetriaminepentaacetate, D-penicillamine, tetraethylenetetraamine (TETA) or trientine, nitrilotriacetic acid (NTA), deferoxamine (DFO), and deferiprone (L1). BAL was the first reported antidote with two sulfhydryl and hydroxyl groups to arsenical nerve gas [175]; the heavy metal binds to a thiol group, resulting in the formation of a stable complex and excretion from the kidney [196]. DMPS is a water-soluble analog of dimercaprol with fewer side effects than dimercaprol [197] and is mainly used to alleviate arsenic and mercury poisoning [198]. DMSA is an analog of dimercaprol with a sulfhydryl group and is a water-soluble, non-toxic, orally administered metal chelator that has been used as an antidote for heavy metal toxicity since the 1950s [199]. Other DMSA analogs have been developed to increase the excretion efficacy [200]. CaNa2EDTA is a water-soluble chelator in which the calcium atom is replaced by lead ions to form a water-soluble complex that is excreted from the kidney and is mainly used for treating lead toxicity [194]. Penicillamine is often used to chelate copper [201]. TETA and NTA are major chelators of copper [202]. DFO is an organic substance that binds tightly to trivalent ions and is used to treat aluminum toxicity [203]. Deferiprone is a major chelator of iron [196].

#### 4.1.2. Stoichiometric Antioxidants

Oxidative stress is one of the primary contributing mechanisms in metal toxicity, offering a strong rationale for the exploration of antioxidant therapy as a treatment for heavy metal toxicity. Free radical scavenging (chain-terminating) antioxidants, such as vitamins E and C, are chemicals with large resonance-stabilized electron clouds that work as electron donors [204] and vitamin C works through the activation of biological antioxidant defenses, such as GSH-linked enzymes [205,206]. Astaxanthin also prevents oxidative stress induced by cadmium [207], copper [208], and cobalt [209] and alleviates cell damage caused by these metals. Selenium [210], and lycopene [211] have been reported as antioxidants against the toxicity of heavy metals, such as cadmium, lead, and chromium.

Other agents have also been reported to be effective in protecting against exposure to heavy metals. The use of probiotic yogurt may be an effective and affordable approach for combating toxic metal exposure through the protection of indigenous gut microbiota in humans, resulting in a faster decrease in copper (34.45%) and nickel (38.34%) levels in the blood [212]. Gossypin, a flavonoid glycoside originally isolated from *Hibiscus vitifolius*, has antioxidant and anti-inflammatory activities and has shown considerable promise for improving recovery in animal models [213].

### 4.2. Pretreatment Drugs

#### 4.2.1. Antioxidants

Pretreatment with several antioxidants is effective in preventing heavy metal overload through direct detoxification of ROS and introduction of some antioxidant enzymes. Melatonin acts as an antioxidant and prevents cadmium and lead overload [214,215]. Coenzyme Q10 works as a NF-E2-related factor 2 (Nrf2) activator and antioxidant [213] Quercetin is a free radical scavenger that can alleviate oxidative stress and protect against lead-induced ER stress by modulating the phosphatidylinositol-3 kinase (PI3K)/protein kinase B (PKB, AKT) and inositol requiring 1 (IRE1)/c-Jun amino-terminal kinase (JNK) pathways in rat liver [216].

Curcumin, a bioactive substance found in turmeric, is widely used as a dietary supplement and has promising metal toxicity-ameliorating effects related to its intrinsic antioxidant activity [217]. Sinapic acid ameliorates cadmium-induced nephrotoxicity involving oxidative stress, apoptosis, and inflammation via NF-κB downregulation [218].

Mitochondrial protection is another target for protection against heavy metal toxicity. Melatonin reduces mitochondrial fission and prevents cadmium-induced cell damage [219]. Metformin also functions as a mitochondrial protection agent by reducing mitochondrial fission and fragmentation and suppressing lead-induced [220] and cadmium-induced toxicity [221].

#### 4.2.2. Senolytic Drugs

Senolytic drugs that induce apoptosis in senescent cells effectively protect against heavy metal toxicity [222]. Senolytic drugs are classified as BCL family inhibitors, PI3K/AKT inhibitors, or FOXO regulators [223,224]. Heavy metals induce phosphorylation of Bcl-2 through activation of the JNK pathway, which is not associated with G2/M cell cycle arrest; hyperphosphorylated Bcl-2 protein can inhibit zinc-induced cell death compared to the hypo-phosphorylated mutant form, indicating that the regulation of Bcl-2 by phosphorylation is an important part of the cell response to heavy metal-induced stress [225]. ROS and heavy metal exposure activate AKT via the PI3K-dependent pathway [226].

Multiple molecules investigated in anti-aging research have been identified as senolytic drugs. Resveratrol prevents cadmium, lead, and manganese toxicity via the JNK or AKT pathways [227,228,229]. Fisetin is a natural flavonoid found in fruits and vegetables [230] and elicits senolytic activity through the AMPK/SIRT1, autophagy, mitochondrial apoptosis, and Rho GTPase signaling pathways, alleviating lead- and mercury-induced toxicity [231]. Mitoquinone (MitoQ) is an antioxidant molecule that targets mitochondrial ATP production and restores mMP, inhibiting lead-induced toxicity [232].

**Table 1:** List of Compounds alleviating heavy metal toxicity.

Compound	Dose	Model	Mechanism	Other Information	References
Vitamin C	500 mg/L drinking water 20–40 mg/kg	Cadmium-treated rat	Oxidative stress	Increases SOD and glutathione peroxidase, and regulates StAR gene expression	Gupta et al. [233], El-Neweshy et al. [234], Ayinde et al. [235]
Vitamin E	100–300 mg/kg, 100 IU/kg	Cadmium-treated rat or rabbit	Oxidative stress Nrf-2 pathway	Increases SOD and glutathione peroxidase and regulates StAR gene expression. Decreases Bax and caspase-9 genes and increase Mfn1, Mfn2, and Bcl-2 Activates the Nrf-2 pathway.	Gupta et al. [233], Amanpour et al. [236], Fang et al. [237], El-Boshy et al. [238], Chen et al. [239], Beytut et al. [240], Ayinde et al. [235]
	80 μg/mL	Lead-treated PC12 cells	Oxidative stress	Decreases ROS levels.	Yang et al. [241]
L-carnitine	100 mg/kg	Lead-treated rats	Oxidative stress	Reduces MDA and elevates TAC levels.	Abdel-Emam et al. [242]
	10–100 mg/kg	Cadmium-treated rats or mice	Oxidative stress	Increases SOD, GSH, and CAT levels.	Iftikhar et al. [211], Abu-El-Zahab et al. [210]
	1 mM	Nickel-treated Neuro-2a cells	Mitochondrial function	Decreases ROS and MDA levels, maintains the mitochondrial membrane potential, and increases mitochondrial DNA copy numbers and transcript levels.	He et al. [243]
Folic acid	0.4 mg/kg	Lead-treated rat	Oxidative stress	Downregulates Bc1-2 and upregulates Bax levels.	Quan et al. [244]
Astaxanthin	10 mg/kg	Cadmium-treated mice	Oxidative stress	Increases CatSper1 and decreases DNA fragmentation.	Saberi et al. [207]
	100 mg/kg	Copper-treated rat	Oxidative stress	Increases G6PD, GR, and GST levels.	Bayramoglu et al. [208,245]
	0.001–10 μM	Copper-treated RWPE-1 cells	Oxidative stress	Decreases MDA and increases MMP, SOD, GSH, and CAT levels.	Meng et al. [246]
	1–20 μM	Cobalt-treated MG-63 cells	Oxidative stress	Regulates Bcl-2 and JNK.	Li et al. [209]
Selenium	0.87 mg/kg	Cadmium-treated mice	Oxidative stress	Increases SOD and CAT levels.	Abu-El-Zahab et al. [210]
Lycopene	4 mg/kg	Cadmium-treated rats	Oxidative stress	Increases SOD, GSH, and CAT levels.	Iftikhar et al. [211]
Fisetin	25 mg/kg or 50 mg/kg	Lead-treated mice	AMPK/SIRT or autophagy pathway	Inhibits apoptosis, decreases Aβ, and increases Aβ removal through neprilysin in the brains of mice.	Yang et al. [247].
	30 mg/kg	Mercury-treated mice	Mitochondrial apoptosis Rho GTPase signaling	Prevents cytochrome c release, reduces ERK 1/2 and caspase 3 levels, and increases RhoA/Rac1/Cdc42 levels in the hippocampus.	Jacob et al. [248]
Quercetin	25–50 mg/kg	Lead-treated rat	IRE1/JNK and PI3K/Akt pathway	Decreases ROS and increases PI3K and PKB/Akt activity.	Liu et al. [249]
Curcumin	15–400 mg/kg	Cadmium-treated mice or rats	Oxidative stress	Increases GSH, CAT, SOD and decrease MDA level.	Eybl et al. [250], Deevika et al. [251], Tarasub et al. [252], Akinyemi et al. [253], Kim et al. [254], Aktas et al. [255], Oguzturk et al. [256], Zoheb et al. [257], Algasham et al. [258], Sharm a et al. [259], Kukongviriyapan et al. [260]
	30–200 mg/kg	Lead-treated mice or rats	Oxidative stress Erk1/2 and JNK pathway	Increases GSH, CAT, and SOD and decreases MDA level.	Abubakar et al. [261,262], Changlek et al. [263], Zahid et al. [264], Alhusaini et al. [265], Soliman et al. [266], Dairam et al. [267]
	5 μM	Copper-treated SH-SY5Y cells,	Oxidative stress	Increases SOD and decrease MDA level. Upregulates pro-caspase 3, pro-caspase 9, and PARP1 expression.	Xiang et al. [268],
	30 mg/kg	Copper-treated rat	Oxidative stress	Increases SOD and GSH levels. Deceases NF-κB and activates the BCl-2/Bax pathway.	Abbaoui et al. [269,270], Yan et al. [271]
Coenzyme Q10	20 mg/kg	Cadmium-treated rats	Oxidative stress	Increases SOD, GSH, and CAT levels.	Iftikhar et al. [211], Paunovic et al. [272]
	10 mg/kg	Lead-treated rats	Oxidative stress Nrf2/HO-1 pathway	Reduces Bax and caspase-3 and increases Bcl-2 levels.	Al-Megrin et al. [273], Yousef et al. [274], Mazandaran et al. [275]
	10 mg/kg	Chromium-treated mice	Nrf2/HO-1/NQO1 pathway	Reduces LPO, GSH, TT, CAT, and PCC and increase SOD and GST levels.	Tripathi et al. [276]
*xanthophylls*	2.5–20 μM	Cobalt-treated Muller cells	Apoptosis and autophagy pathway	Increases Bcl-2 and decreases Bax, cleaved caspase-3, and LC3II levels.	Fung et al. [277]
Resveratrol	20 mg/kg	Cadmium- and lead-treated albino mice	Akt cascade pathway	Reduces p-Akt and increases GSH.	Mitra et al. [227,278]
	400 mg/kg	Cadmium-treated birds	Nuclear xenobiotic receptor response and PINK1/Parkin-mediated Mitophagy	Restores VDAC1, Cyt C, and Sirt3 upregulation and Sirt1, PGC-1α, Nrf1, and TFAM transcription restrictions.	Zhang et al. [279]
	1–100 μM	Cadmium-treated PC12 cells, TCMK-1 cells, MC3T3-E1 cells, ovine oocyte	PP2A/PP5-mediated Erk1/2 and JNK pathway and mTORC1-mediated S6K1/4E-BP1 pathway. ROS decrease and F actin assembly.	Inhibits phosphorylation of S6K1/4E-BP1, Akt, Erk1/2 and/or JNK/c-Jun and cleavage of caspase-3. Increases SIRT1, SOD1, GPX1.	Liu et al. [280,281], Fu et al. [282], Mei et al. [283], Piras et al. [284]
	50 mg/kg	Lead-treated mice or rats	Autophagy pathway Neuroprotective pathway	Inhibits LC3 and Beclin-1 expression and promotes the Aβ degradation and Tau phosphorylation. Increases BDNF and SIRT1.	Bai et al. [228]. Wang et al. [285], Feng et al. [286]
	5 and 10 μM	Copper-treated fibroblast	Autophagy pathway	Reduces carbonylated and polyubiquitinated proteins.	Matos et al. [287]
	10–40 μM	Nickel-treated BEAS-2B cells	P38 MAPK, NLRP3, and NF-κB pathway.	Suppresses p38 MAPK, NF-κB signaling, and NLRP3.	Cao et al. [288]
	10–30 μM	Manganese-treated PC12 cells or rat primary astrocytes	SIRT1 FOXO3a pathway ERK-MMP-9 pathway	Activates SIRT and FOXO3 decreases Bax. Suppresses ERK activity and decreases MMP-9.	Zhao et al. [229], Sun et al. [289], Latronico et al. [290]
	30 mg/kg	Manganese-treated mice	Mitochondrial fragmentation	Activates the deacetylase activity of SIRT1, reduces PGC1α, and regulates DRP1.	Lei et al. [291], Lang et al. [292]
	50 μM	Cobalt-treated cochlear hair cell	Sirtuin1 and NF-κB deacetylation	Activates sirtuin1 and deacetylates NF-κB.	Wang et al. [293]
*Lycium barbarum*	200, 400, and 600 mg/kg	Lead-treated mice	Oxidative stress and apoptosis	Decreases Nrf2 levels.	Xie et al. [294]
	10, 33.3, and 100 mg/kg or 300 mg/kg	Cadmium-induced mice	Oxidative stress	Increases SOD and GSH-Px levels.	Zhang et al. [295], Varoni et al. [296,297].
MitoQ	500 μM	Lead-treated rats	Mitochondrial ATP production and mitochondrial membrane potential	Decreases caspase 3 and 9 activities, synaptosomal lipid peroxidation, and protein oxidation.	Maiti et al. [249]
Melatonin	1 μM	Cadmium-treated HepG2 cells	Autophagy pathway, mitochondrial fission	Inhibits SIRT2-SOD activity and suppresses autophagy. Suppresses the SIRT1-PGC-1α pathway.	Pi et al. [219], Dong et al. [298]
	5–15 mg/kg	Cadmium-treated rats, hamsters, or mice	Oxidative stress, lipid peroxidation	Increases SOD, GSH, decrease MDA levels.	Kim et al. [28], Karbownik et al. [214], Eybl et al. [250], El-Sokkary et al. [25,299], Ji et al. [300],
	10 μM	Lead-treated SH-SY5Y cells	Oxidative stress	Increases GSH levels and inhibits caspase3 activation	Suresh et al. [215]
	10 mg/kg	Lead-treated rat	Oxidative stress, lipid peroxidation	Increases SOD and GSH levels.	El-Sokkary et al. [301]
Rapamycin	0.2 μg/mL, 100 nM–5 μM	Cadmium-treated rat pheochromocytoma (PC12) cells, human neuroblastoma SH-SY5Y cells, human placental trophoblasts, renal tubular cells.	mTORC1 and mTORC2 pathway, mitochondrial ROS-dependent neuronal apoptosis.	Downregulates Akt, S6K1, 4E-BP1. Reduces PP2A and suppresses the activation of JNK and Erk1/2 pathways.	Xu et al. [302,303], Yuan et al. [304], Zhu et al. [305], Kato et al. [306], Chen et al. [307], Fujiki et al. [308], Lee et al. [309]
	5 μM	Lead-treated rat proximal tubular (rPT) cells.	Autophagy pathway	Decreases LC3-II protein levels	Chu et al. [49]
	1.5 mg/kg	Zinc-treated rats	mTOR pathway	Decreases mTOR/p70S6K and increases Nrf2/HO-1.	Lai et al. [310]
	10–100 nM	Copper-treated chicken hepatocytes or RAW264.7 cells	Mitophagy through the PINK1/Parkin pathway, Akt/AMPK/mTOR pathway	Decreases p53, Bak1, Bax, Cyt C, and caspase3/cleaved-caspase3 mRNA and protein levels; increase Bcl2 mRNA.	Yang et al. [311,312], Luo et al. [313]
	0.25 mg/kg	Iron-treated rats	Autophagy pathway	Decreases the ratio phospho-mTOR/total mTOR and restores LC3 II levels.	Uberti et al. [314]
	500 nM	Cobalt-treated HT22 cells or A transformed cell line (RGC-5 cells) with some ganglion cell characteristics	Autophagy pathway	Promotes Beclin-1 expression, increases the conversion of LC3-I into LC3-II, and decreases Bax expression.	Zimmerman et al. [315], Olmo-Aguado et al. [316]
	5 mg/kg	Manganese-treated mice	Autophagy pathway	Activates autophagy and decreased a-Syn oligomers.	Liu et al. [317]
Metformin	2 mM or 250 mg/kg	Lead-treated SH-SY cells or lead -induced rats	Mitochondrial fragmentation, methylglyoxal scavenger.	Increases AMPK/Nrf2 activation. Decreases methylglyoxal and D-lactate.	Yang et al. [220], Huang et al. [318]
	5 mM	Nickel-treated BESA-2B cells	AMPK pathway	Suppresses hexokinase 2 and activates lipocalin 2.	Kang et al. [319,320]
	1 mM	Cadmium-treated spiral ganglion neuron	Autophagy pathway, ROS-dependent PP5/AMPK-JNK signaling pathway	Suppresses LC3-II and p62. Suppresses JNK, and inactive PP5 and AMP.	Li et al. [221], Chen et al. [321]
	100 mg/kg	Cadmium-treated mice	Mitochondrial fission	Decreases Drp1and RB.	Zhang et al. [322]
N-acetyl cysteine	5 mM	Copper-treated GC-1 spg cells	AMPK-mTOR pathway	Reverses mTOR suppression induced by copper.	Guo et al. [323]
*Ganoderma lucidum*	0.1, 0.5, or 1.0 g/kg	Cadmium-treated mice	Metallothionein	Increases metallothionein protein levels and inhibits oxidative stress.	Jin et al. [324]
	0.25 g/kg	Cadmium-treated rats	Oxidative stress and proinflammatory cytokines	Increases SOD, CAT, and GSH levels. Reduces TNF-α, and IL-1β.	Bin-Jumah et al. [325]

Aβ; Amyloid β, AMPK; Adenosine monophosphate (AMP)-activated protein kinase, a-Syn; α-Synuclein, Bax; B-cell/CLL lymphoma 2 (Bcl-2)-associated X protein, Bcl; B-cell/CLL lymphoma, BDNF; Brain-derived neurotrophic factor, CAT; Catalase, Cyt C; Cytochrome c, DRP; Dynamin-related proteins, ERK; Extracellular signal-regulated kinase, FOXO; Forkhead box O, G6PD; Glucose-6-phosphate dehydrogenase, GPX; Glutathione peroxidase, GR; Gutathione reductase, GSH; Reduced glutathione, GST; Glutathione S-transferase, HO; Heme oxygenase, IL; Interleukin, JNK; Jun amino terminal kinase, LPO; Lipid peroxide, MAPK; Mitogen-activated protein kinase, MDA; Malondialdehyde, Mfn; Mitofusin, MMP; Matrix metalloproteinase, NF-κB; Nuclear factor kappa-light-chain-enhancer of activated B cells, NLRP; Nucleotide-binding oligomerization domain-like receptor pyrin-domain-containing protein, NQO1; Nicotinamide adenine dinucleotide phosphate (NAD(P)H) quinone oxidoreductase, Nrf; Nuclear factor, erythroid 2 (NF-E2)-related factor, PCC; protein carbonyl content, PGC; Peroxisome proliferator-activated receptor gamma (PPARγ) coactivator, PINK; Phosphatase and tensin homolog (PTEN) induced putative kinase, PP; Protein phosphatase, SIRT; Silent information regulator, SOD; Superoxide dismutase, StAR; Steroidogenic acute regulatory protein, TAC; Total Antioxidant Capacity, TFAM; Mitochondrial transcription factor A, TNF; Tumor necrosis factor, TOR; Target of Rapamycin, TT; Total thiols, VDAC; Voltage-dependent anion-selective channel.

## 5. Conclusions and Perspectives

Exposure to heavy metals induces various diseases through multiple molecular pathways, including apoptosis, endoplasmic reticulum stress, and mitochondrial stress, resulting in cell damage. These pathways are also implicated in cellular senescence and aging. Many types of heavy metal exposure have been used in aging models in both cell lines and animals. In addition to traditional chelation therapy for heavy metal toxicity, various antioxidants have been found to be effective in treating damage by heavy metal exposure. Advances in research on heavy metals can provide insights into the development of anti-aging and anti-cancer agents.

## Data Availability

The data presented in this study are openly available in the references.
